# The PREVENT study to evaluate the effectiveness and acceptability of a community-based intervention to prevent childhood tuberculosis in Lesotho: study protocol for a cluster randomized controlled trial

**DOI:** 10.1186/s13063-017-2184-0

**Published:** 2017-11-21

**Authors:** Yael Hirsch-Moverman, Andrea A. Howard, Koen Frederix, Limakatso Lebelo, Anneke Hesseling, Sharon Nachman, Joanne E. Mantell, Tsepang Lekhela, Llang Bridget Maama, Wafaa M. El-Sadr

**Affiliations:** 1 ICAP, Mailman School of Public Health, Columbia University, 722 West 168th St, MSPH Box 18, New York, NY 10032 USA; 2 Department of Epidemiology, Mailman School of Public Health, Columbia University, 722 West 168th St, MSPH Box 18, New York, NY USA; 3 ICAP in Lesotho, Columbia University, Lancers Inn, Kingsway and Pioneer Road, Maseru, 100 Lesotho; 4 Desmond Tutu TB Centre, Department of Paediatrics and Child Health, Faculty of Medicine and Health Sciences, Stellenbosch University, Francie van Zijl Drive, Tygerberg, PO Box 241, Cape Town, 8000 South Africa; 5 Pediatric Infectious Diseases, SUNY Stony Brook University, Stony Brook, NY 11794 USA; 6 HIV Center for Clinical & Behavioral Studies, Division of Gender, Sexuality and Health, at the New York State Psychiatric Institute and Columbia University, 1051 Riverside Drive, Unit 15, New York, NY 10032 USA; 7 National Tuberculosis Programme, The Ministry of Health, P.O. Box 514, Maseru, 100 Lesotho

**Keywords:** Ttuberculosis prevention, Child contact management, IPT initiation, IPT completion, Cluster-randomized trial, Mixed-methods, Iimplementation science, Intervention effectiveness, Intervention acceptability

## Abstract

**Background:**

Effective, evidence-based interventions to prevent childhood tuberculosis (TB) in high TB/HIV-burden, resource-limited settings are urgently needed. There is limited implementation of evidence-based contact management strategies, including isoniazid preventive therapy (IPT), for child contacts of TB cases in Lesotho.

**Methods/design:**

This mixed-methods implementation science study utilizes a two-arm cluster-randomized trial design with randomization at the health facility level. The study aims to evaluate the effectiveness and acceptability of a combination community-based intervention (CBI) versus standard of care (SOC) for the management of child TB contacts. The study includes three phases: (I) exploratory phase; (II) intervention implementation and testing phase; (III) post-intervention explanatory phase. Healthcare provider interviews to inform intervention refinement (phase I) were completed in December 2015. In phase II, 10 health facilities were randomized to deliver the CBI or SOC, with stratification by facility type (i.e., hospital vs. health center). CBI holistically addresses the complex provider-related, patient-related, and caregiver-related barriers to prevention of childhood TB through nurse training and mentorship; health education for caregivers and patients by village health workers; adherence support using text messaging and village health workers; and multidisciplinary team meetings, where programmatic data are reviewed and challenges and solutions are discussed. SOC sites follow country guidelines for child TB contact management. Routine TB program data will be abstracted for all adult TB cases newly registered during the study period and their child contacts from TB registers and cards. The anticipated sample size is 1080 child contacts. Primary outcomes are yield (number) of child contacts, including children < 5 years of age and HIV-positive children < 15 years of age; IPT initiation; and IPT completion. Secondary outcomes include HIV testing; yield of active prevalent TB among child contacts; and acceptability and utilization of CBI components. Intervention implementation began in February 2016 and is ongoing. Post-intervention interviews with healthcare providers and caregivers (phase III) commenced in February 2017.

**Discussion:**

The PREVENT study tests the effectiveness and acceptability of a novel combination CBI for child TB contact management in Lesotho. If effective, CBI will have important implications for addressing childhood TB in Lesotho and elsewhere.

**Trial registration:**

ClinicalTrials.gov, NCT02662829. Registered on 15 January 2016.

**Electronic supplementary material:**

The online version of this article (doi:10.1186/s13063-017-2184-0) contains supplementary material, which is available to authorized users.

## Background

The tuberculosis (TB) epidemic has had an enormous global impact, particularly in sub-Saharan Africa. The World Health Organization (WHO) estimated that in 2015 there were 10.4 million new TB cases and 1.8 million deaths from TB [1]. Worldwide, among people with HIV, TB is the most common opportunistic illness and the leading cause of death [2, 3]. Over 1 million children are estimated to develop TB every year [1] and 67 million are estimated to be infected with *Mycobacterium tuberculosis* [4] with more than 8 million infected annually [5]. Children in resource-limited settings with high HIV and TB rates suffer an enormous, but often unappreciated, TB disease burden [6]. Children less than 15 years of age are estimated to contribute between 10% and 20% of the disease burden in TB-endemic areas [5]. In such areas there has been a marked age and gender shift, with more women developing TB during their reproductive years [7], increasing the likelihood of young children being exposed to TB within the household [8]. Although TB is preventable and curable, it kills at least 210,000 children each year and is among the top 10 causes of death in children globally [1]. Higher mortality highlights the importance of TB prevention, particularly among people with HIV [9] and young children [10], both of whom have an increased risk of developing TB following infection and a higher risk of disseminated or severe disease [11].

Child TB contact management includes identifying, screening and evaluating child contacts exposed to TB, and initiating and ensuring completion of either isoniazid preventive therapy (IPT) or appropriate treatment for active TB. Numerous studies have found that screening household contacts is effective in preventing TB, through offering IPT for eligible individuals, and in identifying prevalent TB cases [12]. Six to nine months of IPT has proven efficacy to prevent TB in persons with HIV infection and recent close contacts [13]. A recent meta-analysis of IPT efficacy in preventing TB in children identified a 59% reduction in risk among children < 15 years of age, excluding children < 4 months of age [14].

The World Health Organization (WHO) recommends that children under the age of 5 years and HIV-positive children who are contacts be identified and screened for TB symptoms and IPT be offered to eligible children; children with presumptive TB should be investigated and initiated on TB treatment, as appropriate [15]. In 2015 there were an estimated 1.2 million children who were household contacts of patients with bacteriologically confirmed pulmonary TB and who were eligible for TB preventive treatment according to current policy recommendations [1]. Despite these guidelines, child TB contact management has not been routinely or effectively implemented in resource-limited settings, where most childhood TB occurs, and is characterized by low attendance for screening, poor adherence to IPT, and high lost-to-follow-up rates [16, 17]. A recent systematic review in countries with a high TB burden demonstrated that at each step in the child TB contact management cascade, opportunities were missed to prevent TB [18]. Innovative, evidence-based approaches are needed to strengthen identification and management of child contacts in areas of high TB incidence, as these children can benefit from both IPT and case finding via tracing and screening.

Lesotho, a lower-middle-income sub-Saharan African country with a population of 2.1 million [19], has the world’s second highest TB incidence, estimated at 788 per 100,000, with approximately 72% of patients with TB co-infected with HIV [1]. HIV prevalence—at 24.6%—is the world’s second highest [20, 21]. Available data from other countries in settings with poor epidemic control suggest that TB incidence in children is likely to be 50% of adult TB incidence in such contexts [6]. In Lesotho, 5–7% of TB cases are reported in children [1, 22], which is low compared to similar settings such as in neighboring South Africa, where the proportion of pediatric cases is at least 10–20% [23]. This lower-than-expected reporting of pediatric cases is most likely due to under diagnosis of TB in children in Lesotho. In 2011, the Lesotho National TB Program adopted the WHO child TB contact management recommendations. However, as in other countries in the region, implementation is limited, with no well-defined strategies guiding child TB contact management and no clear methods enabling provision of IPT to children [24, 25]. Thus, it is important to evaluate novel strategies to ensure delivery of TB prevention services in child contacts.

While several studies have evaluated the effect of an individual intervention on one step of the child TB contact management cascade, few have evaluated a combination approach that holistically addresses the complex provider-related, patient-related, and caregiver-related barriers reported in the literature [24–32]. Furthermore, there is a need for implementation science research to test the proposed combination strategy in a pragmatic way; evaluate its acceptability among healthcare providers, patients, and caregivers; and provide information on the implementation process itself [33].

We describe the design of the PREVENT study, a mixed-methods cluster-randomized implementation science study that aims to evaluate the effectiveness and acceptability of a combination community-based intervention (CBI) vs. standard of care (SOC) to identify and screen child contacts of adults with TB in Berea District, Lesotho, and provide those eligible with IPT.

## Methods

### Study design

PREVENT is a mixed-methods cluster-randomized implementation science study that includes three implementation phases:

1. Phase I: a pre-intervention exploratory phase that allows for intervention refinement by assessing training needs and soliciting feedback on planned intervention components from healthcare providers.
2. Phase II: an intervention implementation and testing phase utilizing a cluster-randomized trial design, with randomization at health facility level, to evaluate the effectiveness of CBI versus SOC to identify and screen child contacts of adult TB cases and to provide IPT to eligible children.
3. Phase III: a post-intervention explanatory phase utilizing in-depth interviews with key informants (healthcare providers and caregivers) at CBI sites to assess acceptability and utilization of intervention components as well as the overall combination strategy.

### Study setting and context

The study is conducted in Berea District in Lesotho. Childhood TB in Lesotho is largely managed by TB clinic nurses in health facilities, who provide outpatient care and preventive services, with complicated cases referred to district hospitals. Typically, each TB clinic is staffed by one to three nurses and one or two lay counselors, who are supported by 20–30 community-based village health workers (VHWs). The Ministry of Health (MOH) supplies each health facility with isoniazid 100 mg tablets for the prevention of TB in child contacts as per standard of care; isoniazid suspension is not available in Lesotho. Isoniazid and vitamin B6 (as adjunctive to IPT) are dispensed in on-site pharmacies at health facilities.

The PREVENT study is supported by the MOH as it is aligned with its strategies and priorities. Individuals from MOH are represented on the research team and on the study’s Stakeholder Advisory Group. The National TB Program Manager and Berea District Health Management Team Manager were engaged early, at the time of intervention design, to ensure that study findings inform national policy and programming.

### Facility selection

In collaboration with the MOH, a single district was chosen for conduct of the study in order to enhance internal validity and maximize implementation cost efficiency. Of 19 public health facilities that provide TB services in Berea District, 10 were selected as study sites. The remaining nine health facilities were excluded from the sampling frame because of low TB patient case load (on average, < 6 patients with TB notified per quarter). Both hospitals (*N* = 2) and health centers (*N* = 8) were included to enhance generalizability of study findings as TB services are provided in both types of facility in Lesotho.

### Assignment to study arm

As this is a cluster-randomized trial, assignment to study arm was done at the health facility level and not at the individual participant level. Ten health facilities (clusters) were randomized to deliver CBI or SOC, following stratification by facility type (hospital or health center). Before randomization, study sites were numbered sequentially within each stratum and intervention status was randomly assigned within each stratum by the Principal Investigator (PI) using SAS v. 9.3 (SAS Institute, Cary, NC, USA). Patients enrolled in health facilities assigned to the SOC arm receive standard of care supported by the Lesotho MOH, whereas those enrolled in health facilities assigned to the CBI arm receive the SOC plus the community-based intervention. Healthcare providers, patients, and study staff are not blinded to the assigned study arm.

### Study participants

#### Newly registered adult patients with TB and their child contacts

Routinely collected data are to be abstracted for all adult patients with TB newly registered for TB treatment during the study period (3 years) and their child contacts at the 10 participating health facilities.

#### Key informants

Two groups of key informants from CBI sites are targeted for the study: (1) healthcare providers and (2) caregivers. Eligibility criteria for healthcare providers are: (1) nurse or facility-based lead VHW (LVHW) working in a CBI site or VHW working in the community and affiliated with the CBI site; (2) aged 18 years or older; (3) English-speaking or Sesotho-speaking; and (4) capable of providing informed consent.

Eligibility criteria for caregivers are: (1) caregiver of a child contact in a CBI site; (2) aged 18 years or older; (3) English-speaking or Sesotho-speaking; and (4) capable of providing informed consent. Both caregivers who brought their children for TB screening and caregivers who did not bring their children for TB screening will be targeted for enrollment.

### Interventions

#### Standard of care (SOC)

At health facilities randomly assigned to SOC, patients receive usual care for child TB contact management, which includes contact tracing, screening, and IPT provision. As per national guidelines, adult patients with TB are asked to bring child contacts to the TB clinic, where they are screened for TB using a simple symptom questionnaire [34]. Sputum, when available, is sent for smear microscopy in children with a positive symptom screen; children requiring sputum induction and all HIV-positive children under one year of age are referred to the district hospital. Gastric aspirates are not conducted in Lesotho [34]; Xpert MTB/RIF, culture, and drug susceptibility testing are only requested in retreatment cases or children exposed to multidrug-resistant TB. Chest radiographs are performed at the district hospital for symptomatic children with a negative sputum smear; tuberculin skin testing and interferon gamma release assays are not available. Children with a positive sputum smear or chest radiograph are treated for active TB, and those screening negative are assessed for IPT eligibility. After excluding those with contraindications for IPT (e.g., acute or chronic liver disease, symptoms of severe peripheral neuropathy, and kidney failure), nurses counsel children and caregivers on IPT benefits, potential side effects, and the importance of adherence. Daily isoniazid and vitamin B6 are prescribed according to the child’s weight, and caregivers are instructed to crush the tablets and mix with food so that they can be easily swallowed. Nurses enter all child contacts’ information into contact tracing registers and open a facility-based IPT card for each child contact who initiates IPT.

After IPT initiation, patients and caregivers are encouraged to return to the health facility monthly for monitoring of side effects, TB symptoms, and adherence, and to be provided with a 30-day supply of isoniazid and vitamin B6. The dosage of isoniazid and B6 is adjusted, if indicated, according to the child’s weight at every visit. If adherence problems are noted, the nurse is expected to counsel the patient and caregiver. The tracing of patients lost to follow up is usually passive and such efforts are inconsistent across facilities.

VHWs are expected to provide treatment support to adult patients with TB, but have a limited role in supporting caregivers and child contacts and have infrequent contact with providers at nearby health facilities. Additionally, there is no standardized, nationally approved curriculum for educating patients and caregivers about TB treatment, IPT, and adherence literacy.

#### Community-based intervention (CBI)

In the exploratory phase of PREVENT (phase I), we used a participatory approach to intervention development [35] by conducting group interviews with healthcare providers at CBI sites before the intervention launch. We explored attitudes toward TB prevention, assessed training needs, and solicited feedback on planned intervention components to be introduced as part of the CBI. Providers requested additional training, especially on TB diagnosis, and found the proposed study job aids (see subsequent description) to be useful tools that would assist them with proper patient management. Providers recommended creating additional health education materials such as TB prevention wall posters to post in the clinic waiting room and brochures that could be distributed during community outreach activities. The intervention was refined based on findings from the exploratory phase.

At health facilities randomly assigned to CBI, the combination intervention is delivered to all adult TB cases and their child contacts and caregivers. All nurses and LVHWs were trained to implement the CBI that holistically addresses the complex provider-related, patient-related, and caregiver-related barriers to prevention of childhood TB using job aids. CBI includes: (1) mentoring of nurses and LVHWs in child TB contact management so as to enable them to inform index cases and caregivers about the potential benefits of TB prevention; (2) visits by VHW to all household contacts of adults with TB, and referral of all children < 5 years of age regardless of HIV status, and all HIV-exposed and HIV-positive infants and children < 15 years of age to health facilities; (3) assessment of child contacts by nurses and intensive adherence education and support to caregivers by LVHWs using study-developed job aids; IPT is initiated for eligible children, after exclusion of active TB as per SOC in Lesotho; (4) adherence support via weekly short message service (SMS) medication reminders and monthly SMS appointment reminder messages sent by LVHWs and VHW support in the community; and (5) monitoring and review of data on IPT initiation and adherence in quarterly multidisciplinary team meetings to inform problem-solving and corrective action. Table 1 shows a comparison of study arms.

**Table 1:** Comparison of study arms

	Standard of care (SOC)	Community-based intervention (CBI)
Adult patients asked to bring child contacts to TB clinic for screening	X	X
Child contacts screened with symptom questionnaire	X	X
IPT offered to eligible child contacts	X	X
Monthly IPT visits	X	X
HIV testing encouraged for eligible child contacts	X	X
Active community contact tracing		X
Community-based health education using study brochure		X
Child screening and IPT provision according to clinical algorithm		X
Nurse training and ongoing mentorship		X
Health education for caregivers using treatment literacy curriculum		X
Active tracking of IPT provision		X
Consistent community support via VHW		X
Social support and navigation by VHW		X
SMS medication and appointment reminder messages		X
Review of IPT monitoring data at quarterly multidisciplinary team meetings		X

*TB* tuberculosis, *IPT* Isoniazid preventive therapy, *VHW* village health worker, SMS short message service

Nurses use a clinical algorithm developed for the study based on national guidelines for intensive case finding and screening of child contacts to assess patients without TB symptoms for IPT eligibility, to initiate IPT, and to monitor side effects, TB symptoms, and adherence. If a child contact develops TB symptoms during IPT, national guidelines are followed for further investigation and management. Nurses also use a laminated study poster of dosage tables for isoniazid and vitamin B6 to calculate appropriate dosages. The study nurse mentor emphasizes to nurses the importance of using the MOH’s contact tracing registers and IPT cards, and HIV testing in child contacts. Nurses explain to caregivers that IPT can prevent TB, promote IPT initiation, assess IPT adherence and side effects, and encourage follow up with the VHW. Nurses also emphasize the importance of HIV testing for child contacts.

LVHWs use a scripted, illustrated flipchart to educate caregivers and patients on the importance of TB prevention, IPT provision, and adherence. LVHWs provide real-time adherence support via SMS messages and follow up with caregivers of children who miss appointments or report nonadherence. LVHWs offer support, provide referrals, and advocate for patients.

Nurses and LVHWs separately attended two half-day training sessions on study interventions. Quarterly refresher training and weekly mentorship are provided to nurses and LVHWs on-site by a study nurse mentor. All nurses and LVHWs from CBI sites meet as a team quarterly to review IPT data and intervention activities, identify challenges, and develop solutions; a small motivational reward (500 loti, approximately US$36) is awarded to the best performing site in the previous quarter. The best performing site is selected by the TB/HIV coordinator of the Berea District Health Management Team and the study nurse mentor based on high proportions of IPT initiation and completion, high proportions of HIV testing, good documentation of contact management activities, the LVHW’s coordination and accountability, and timely submission of reports.

To investigate household contacts, VHWs visit the homes of all adults with TB registered at facilities assigned to CBI. All HIV-positive and HIV-exposed children and children < 5 years of age, regardless of HIV status, are referred to health facilities. VHWs administer TB symptom screening to child contacts in the community, accompany them and their caregivers to the clinic, and provide education sessions and adherence counseling. In addition, LVHWs conduct community education sessions on TB prevention using an illustrated brochure developed for the study. A wall poster, developed for the study is displayed at every CBI site to reinforce the importance of TB prevention in children to caregivers and patients.

### Primary and secondary endpoints

While the facilities are randomized by site, all outcomes are determined at an individual participant level. Phase II primary outcomes are: (1) overall yield of child contacts defined as the number of child contacts screened per adult TB case diagnosed during the study period; (2) appropriate IPT initiation defined as the proportion of child contacts identified through contact tracing of new adult patients with TB during the study period, who are determined to be eligible for and who initiate IPT, based on review of clinic records; and (3) IPT completion defined as the proportion of child contacts, who complete 6 months of daily IPT, out of those who initiate IPT, as determined by healthcare provider and recorded in clinic records (Table 2).

**Table 2:** Study outcomes

Study outcome	TB index patients	Child contacts	Key informants - caregivers	Key informants - healthcare providers
Yield of child contacts^a^	X			
Screened		X		
IPT initiation^a^		X		
IPT completion^a^		X		
Yield of TB		X		
HIV testing		X		
Acceptability of intervention components			X	X
Reasons for IPT non-initiation			X	

*TB* tuberculosis, *IPT* Isoniazid preventive therapy

^a^Primary outcomes

Phase II secondary outcomes include: (1) HIV testing defined as the proportion of child contacts identified through contact tracing of new adult patients with TB during the study period, who are tested for HIV, based on review of clinic records; and (2) yield of active prevalent TB in child contacts defined as the number of child contacts diagnosed with active TB per adult TB case

The phase III secondary outcome is the acceptability of the intervention to caregivers and healthcare providers, which will be characterized via in-depth qualitative analysis and interpretation [36].

### Sample size and power calculations for phase II

Power calculations were based on the primary outcomes of yield of child contacts and IPT initiation and completion. Based on previous programmatic data from the setting, we anticipated an average of 54 new TB cases per facility per year in Berea District. Based on past experience, the Lesotho TB program estimates that the number of child contacts currently identified per case is 0.5. Thus, at least 27 child contacts are expected to be screened for TB symptoms per SOC facility per year. Based on previous programmatic data, an estimated 5% of contacts will be found to have active TB and fewer than 5% will have contraindications for IPT, resulting in at least 25 child contacts eligible for IPT per facility per year for a total of 75 IPT eligible child contacts per facility over the 3 years of the study. CBI is hypothesized to increase identified child contacts from an average of 0.5 to 2.0 for each adult TB case. Using the two-sided *t* test with a significance level of 0.05 and a standard deviation of 2.5, we will have 91% power to detect a difference of 1.5 between group means. Assuming the two-sided Farrington and Manning score test with α = 0.05 and an intra-cluster correlation coefficient (ICC) of 0.05, we will have between 79% and 98% power to detect a difference in IPT initiation from 20% (SOC) to 40–50% (CBI). This will result in an estimated total of 375–450 child contacts initiating IPT across all sites. Assuming that 20% of child contacts will initiate IPT, 15 child contacts at each clinic will be eligible for IPT completion. Using the two-sided Farrington and Manning score test with α = 0.05 and ICC of 0.05, we will have 83–99% power to detect a difference in IPT completion from 30% (SOC) to 60–70% (CBI).

### Recruitment

All potential key informants are referred to the study by the nurse in charge at each study site. Study staff meets with all potential key informant participants (healthcare providers and caregivers) in a private area to provide further information about the study using a standardized script, assess eligibility, and obtain written informed consent. The study was deemed eligible for waiver of individual consent for index cases and their child contacts (see “Ethics and consent process”). Thus, there is no active recruitment of index cases and their child contacts in this implementation science study as the focus is on medical record review.

Phase I: pre-intervention – healthcare providers: convenience sampling was utilized, with the following recruitment targets: (1) at least one nurse per CBI site; (2) at least one LVHW from a total of three CBI sites.

Phase III: post intervention – healthcare providers: interviews commenced after the intervention has been implemented for at least one year. Convenience sampling is utilized to enroll 15–30 healthcare providers, with the following recruitment targets: (1) at least one nurse per CBI site; (2) at least one LVHW per CBI site.; and (3) at least one community-based VHW per CBI site. On average, five nurses and one to two LVHWs are employed at each site, and additional community-based VHWs are associated with each site. A sample size of 15–30 healthcare providers should enable us to reach data saturation.

Phase III: post intervention – caregivers: heterogeneous purposive sampling [37, 38] is utilized to enroll 30 caregivers according to the following recruitment targets: (1) 80% of caregivers who brought their children for TB screening, and 20% of caregivers who did not bring their children for TB screening; and (2) a proportional number of participants at each CBI site, based on patient intake. In addition, we will try to include caregivers whose children may have struggled with adherence issues. This target sample size will be augmented if we find that data saturation has not been reached based on analyses of the transcripts.

Interviews with caregivers who did not bring their children for TB screening will be conducted after cessation of intervention activities. Based on our prior experience, conducting in-depth interviews with patients (caregivers in this case) who refuse or delay recommended guidelines will be challenging. However, caregivers who did not bring their children for TB screening represent a very important group for targeted TB prevention. We will utilize our community-based VHW to explain that sharing their opinions and perspectives on declining TB screening for their child contacts is of utmost importance so that the MOH can design more appropriate future interventions. In-depth interviews will not be conducted in the community; caregivers will be compensated for their travel costs to the health facility.

### Data collection

Multiple data collection methods are utilized and include: (1) abstraction of quantitative outcome data from clinic records; (2) in-depth qualitative healthcare provider and caregiver interviews; and (3) documentation of process data through facility characteristics surveys and intervention utilization logs.

#### Quantitative data

Individual patient-level data will be collected from medical records using a standardized data abstraction tool. Information collected will include demographic and treatment information on all index cases and child contacts identified and screened for TB, diagnosis of TB and treatment initiated, IPT initiation, IPT completion, and HIV testing.

#### Qualitative data

Open-ended interview guides are used to stimulate discussion with key informants. Organizations with prior experience in translating health and technical information will translate study consent forms and post-intervention interview guides from English into Sesotho. Back-translation from Sesotho into English will be performed to verify translation accuracy and to ensure fidelity to the questions’ original intent.

Phase I interviews were conducted by a trained, experienced, qualitative interviewer with clinic nurses and the LVHW at each CBI site in a private space on-site prior to the start of the intervention; these data were used to modify the intervention. The interview guide consisted of open-ended, exploratory questions that were asked in a non-judgmental and culturally sensitive way to capture emic perspectives. Respect for participants’ privacy and confidentiality was emphasized in group interviews, and divergent perspectives were encouraged.

After intervention implementation for at least one year, additional healthcare provider interviews with nurses and LVHWs, and caregiver interviews are conducted by the same qualitative interviewer to evaluate acceptability and utilization of intervention components and the combination strategy. The Principal Investigator (PI) closely supervises the interviewer and provides timely feedback, particularly regarding probing, not asking leading questions or responding to participants in leading ways, and maintaining affective neutrality in facial expressions and body language.

#### Process data

A structured survey of facility characteristics (e.g., number of providers, stock-outs of medical and laboratory supplies, availability of laboratory services, counseling, clinical algorithms, educational materials) was conducted by the study nurse mentor with the nurse in charge at each facility prior to intervention implementation and is administered quarterly thereafter at all 10 participating health facilities. The survey documents baseline characteristics at each facility and monitors changes in intervention implementation over time. Additionally, at each CBI site, the LVHW completes an intervention log that tracks delivery of SMS messages and education sessions to document the dosage of intervention components received by each patient; the nurse mentor completes mentoring logs that assess intervention quality [33].

### Data management

Data on patients with TB and their child contacts are collected and entered directly on a dedicated study tablet, using a unique study identification number, which is stored in a secure double-locked filing cabinet at the study office. The study database is encrypted and password-protected. Established quality control measures such as skip patterns, range limitations, and consistency checks are incorporated into the database to enhance the accuracy and completeness of the data collected. The database is backed up nightly to an encrypted external hard drive maintained in a locked filing cabinet. Each key informant interview is digitally audio-recorded, transcribed verbatim, and translated to English, if necessary.

### Statistical methods

Phase I: grounded theory methods were used to analyze data from Phase I, a pre-intervention exploratory phase that allowed for intervention refinement.

Phase II: an intent-to-treat analysis will be used. Generalized linear mixed models will be applied to test for differences between study arms for dichotomous (IPT initiation, IPT completion, HIV testing) and continuous (yield of child contacts, yield of active TB) outcomes. Models will include fixed effects for study arm and patient characteristics, and random effects for study site to adjust for potential non-independence of observations.

Phase III: the analysis of acceptability will be based on grounded theory framework, which encourages the emergence of ideas and theories from within the dataset [36, 39]. This will help to elucidate the complex, social pathways that may impact TB prevention efforts among child contacts. An iterative process to data collection and analysis will be used. All in-depth interview audio-recordings and supplementary and contextual notes will be transcribed verbatim and translated from Sesotho into English. A preliminary review of the first five interview transcripts in each group will be conducted to develop an initial codebook that will be subsequently be applied to the remaining interviews. Dedoose software will be used to manage and code data and facilitate systematic data management. A list of broad codes will be compiled, based on preliminary review of transcripts. Codes deemed relevant for the study aims will be re-applied to the transcripts to allow for active development of themes. Coding results will be regularly assessed for inter-rater reliability in coding and text segmentation. Thematic comparisons within and across narratives will be used to identify latent patterns and negative cases in relation to TB prevention issues. Theoretical notions about the role of caregivers in IPT initiation and adherence will be developed by analyzing the study themes in the context of existing literature and theorizations related to TB treatment initiation and adherence. Data from providers and caregivers will be analyzed separately and comparatively. Data analysis will explore contextual factors related to caregivers’ and providers’ perceptions of acceptability and utilization of intervention components and illuminate common and divergent themes.

### Monitoring

As this is an implementation science study utilizing the recommended standard of care child TB contact management as per country guidelines rather than an experimental intervention, a data monitoring committee was not deemed necessary. Study personnel were trained to assess study-related adverse events, such as loss of confidentiality, and to notify the PI immediately if they learned of an adverse event. In the event of an adverse event, an incident report will be completed describing the incident, its possible cause, and steps taken to address the adverse event and to prevent its recurrence. The Columbia University Medical Center Institutional Review Board and the Lesotho National Health Research and Ethics Committee will be informed according to their respective reporting guidelines.

Internal monitoring of intervention delivery at each study site is performed weekly by the nurse mentor, to ensure that each site is adhering to study standard operating procedures; in case of non-adherence, the nurse mentor will retrain the providers. In addition, the district TB/HIV coordinator monitors contact tracing and IPT provision for all study sites. External monitoring visits are performed three times per year by the PI and include review of each site’s performance and adherence to confidentiality guidelines.

### Ethics and consent process

The protocol was reviewed and approved by the Columbia University Medical Center Institutional Review Board (Ref AAAN7358) and the Lesotho National Health Research and Ethics Committee (Ref ID78-2015). Protocol modifications are to be submitted to the ethics committees. Both regulatory entities determined the medical record review as eligible for waiver of individual consent for index cases and their child contacts. Healthcare providers and caregivers who participate in the key informant interviews provide written informed consent. Consent forms and all of the identifying information obtained from study participants are stored in separate locked filing cabinets in a locked room. Upon determination of eligibility, participants are assigned unique identification numbers. The study database includes participant unique identification numbers only; no participant names or identifiers are recorded. A master list with each participant’s name and unique identification number is in a locked cabinet, and will be maintained only long enough to permit study investigators to review and audit the data; afterwards, this document will be destroyed, as per standard approaches. Investigators have and will maintain access to the full trial dataset.

**Fig. 1:**
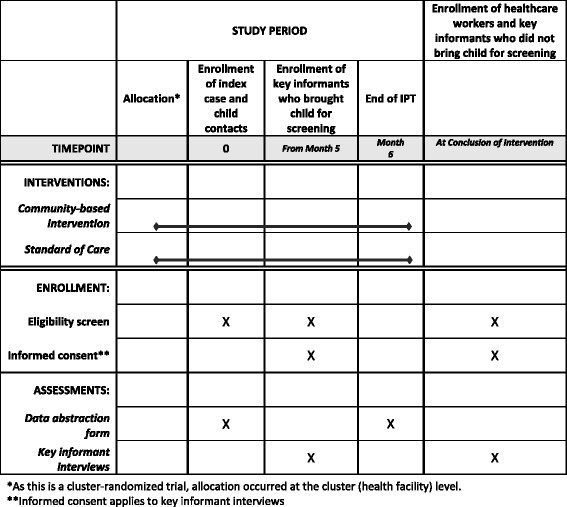
Schedule of study interventions and assessments

The trial design and protocol adhere to Standard Protocol items: Recommendations for Interventional Trials (SPIRIT) criteria (www.spirit-statement.org); see the SPIRIT figure (Fig. 1). The SPIRIT checklist can be found as Additional file 1: Table S1.

### Dissemination

A dissemination strategy was developed to ensure that study findings are shared with key stakeholders, regardless of the magnitude or direction of effect. This strategy includes a dissemination meeting in Lesotho with the MOH, District Health Management Team, healthcare providers, and patients from participating health facilities; a dissemination report for the MOH; dissemination of findings through the WHO Childhood TB subgroup; presentations at scientific conferences; and publications in peer-reviewed journals. Investigators will adhere to recommendations from the International Committee of Medical Journal Editors regarding authorship. Data, which have been stripped of all identifiers, will be made publicly available following the publication of primary and secondary outcome papers in accordance with the National Institutes of Health Data Sharing Policy [40].

## Discussion

We described the PREVENT study, a mixed-methods cluster-randomized implementation science study that aims to evaluate the effectiveness and acceptability of a novel combination community-based intervention strategy versus standard of care to identify and screen child contacts of adult TB cases in Berea District, Lesotho, and provide them with IPT to prevent the development of TB.

The cluster-randomized study design in which study interventions are delivered at the health-facility level was deemed more feasible than an individual-randomized design. This allows clinic staff at each site to provide all of their patients with the same package of interventions rather than varying interventions to different patients. Individual randomization can potentially disrupt service delivery as individuals randomized to SOC may believe they are receiving an inferior strategy, which in turn, can influence their behavior and study outcomes.

The study has several strengths. The cluster-randomized approach permits causal attribution of observed outcomes to the CBI by comparing them to the counterfactual scenario, while reducing selection bias [41]. In addition, the study aims to assess an innovative multicomponent implementation intervention that builds on prior scientific evidence, while addressing the diverse barriers to TB prevention in child contacts. Furthermore, the selected study sites have adequate heterogeneity, covering facilities in both urban and rural locations in low land, foothill and mountainous areas, which will strengthen the external validity of findings. Use of mixed methods [42, 43] in a sequential exploratory design (qualitative data collection and analysis in the first phase followed by collection and analysis of quantitative data) and a sequential explanatory phase (quantitative data collection and analysis followed by collection and analysis of qualitative data), allows us to refine the intervention, test it, and evaluate acceptability and utilization of intervention components and the overall strategy. Finally, stakeholder engagement in all study phases will foster MOH ownership at the national and district levels, and will help to ensure successful integration of study findings in policy and programmatic contexts.

Limitations of the study design include the possibility of participant crossover between CBI and SOC sites, which could potentially decrease power to identify a difference between study arms. An additional risk is the potential for diffusion of the CBI components (clinical algorithm, flip chart, etc.) to the SOC sites during the study period. CBI providers have been asked to refrain from discussing the CBI and not to share job aids with SOC providers. The degree to which any CBI components are implemented at SOC sites during the study period will be measured using process documentation. Additionally, migration and loss to follow up could affect endpoint ascertainment. However, given the goal of assessing CBI effectiveness in a health systems context, it is important to evaluate its impact in realistic program scenarios while monitoring crossover and migration using process documentation. Another potential limitation is unanticipated health system inefficiencies, such as shortages of medications or interruptions in healthcare provider availability, which may impact CBI delivery. However, as the study is conducted in one district and all sites rely on the same supply chain, any changes (e.g. stock-outs) will likely be similar across study arms and reflect system dynamics captured in implementation science. We will track this information using process documentation. The study is relying on routinely collected programmatic data for ascertaining study outcomes, which means that data may be incomplete. However, procedures will be implemented at all study sites that aim to mitigate the amount of missing data. Last, the study design precludes evaluation of the effectiveness of individual components of the CBI. However, qualitative results will highlight provider and caregiver perspectives on the acceptability of various CBI components and similarly process data will demonstrate utilization of these components.

Effective, evidence-based interventions to prevent childhood TB in high TB-burden and HIV-burden, resource-limited settings are urgently needed. In the PREVENT study, innovative methodology is used to assess the effectiveness and acceptability of a combination intervention that holistically addresses the complex provider-related, patient-related, and caregiver-related barriers to prevention of childhood TB. It is hypothesized that using a feasible community-based model of care will improve TB prevention in young, vulnerable children.

The PREVENT study has the potential to advance the science and practice of TB contact management for children in this setting. If effective, it will have important implications for programs and policies within Lesotho, and more broadly for high TB-burden and HIV-burden resource-limited countries in sub-Saharan Africa, where children are particularly vulnerable.

## Study status

The study commenced and completed recruitment of healthcare provider key informants (phase I) in December 2015. Trial activities of intervention implementation and testing (phase II) commenced in February 2016 and are expected to continue through January 2019. In-depth interviews with healthcare providers and caregivers (phase III) commenced in February 2017. The trial is currently recruiting.

## Additional file

Additional file 1: Table S1. SPIRIT 2013 Checklist PREVENT. (DOCX 60 kb)

## Abbreviations

CBI: Community-based intervention
HIV: Human immunodeficiency virus
ICC: Intra-cluster correlation coefficient
IPT: Isoniazid preventive therapy
LVHW: Lead village health worker
MOH: Ministry of Health
NIAID: National Institute of Allergy and Infectious Diseases
PI: Principal Investigator
SMS: Short message service
SOC: Standard of care
TB: Tuberculosis
VHW: Village health worker
WHO: World Health Organization
